# The relationship between tumour necrosis, tumour proliferation, local and systemic inflammation, microvessel density and survival in patients undergoing potentially curative resection of oesophageal adenocarcinoma

**DOI:** 10.1038/bjc.2011.610

**Published:** 2012-01-12

**Authors:** S Dutta, J J Going, A B C Crumley, Z Mohammed, C Orange, J Edwards, G M Fullarton, P G Horgan, D C McMillan

**Affiliations:** 1 University Department of Surgery, Faculty of Medicine, University of Glasgow, Royal Infirmary, Glasgow, UK; 2 Department of Pathology, Glasgow Royal Infirmary, Glasgow, UK; 3 Unit of Experimental Therapeutics, Department of Surgery, Institute of Cancer, College of MVLS University of Glasgow, Western Infirmary, Glasgow, UK

**Keywords:** oesophageal cancer, C-reactive protein, tumour inflammatory infiltrate, microvessel, necrosis, survival

## Abstract

**Background:**

There is increasing evidence that the local and systemic inflammatory responses are associated with survival in oesophageal cancer. The aim of this study was to examine the relationship between tumour necrosis, tumour proliferation, local and systemic inflammation and microvessel density and survival in patients undergoing potentially curative resection of oesophageal adenocarcinoma.

**Methods:**

The interrelationship between tumour necrosis, tumour proliferation, local inflammatory response (Klintrup–Makinen criteria, intra-tumoural CD8+ lymphocyte and macrophage infiltration), systemic inflammatory response (modified Glasgow Prognostic score (mGPS)), and microvessel density was examined in 121 patients undergoing potentially curative resection for oesophageal adenocarcinoma (including type I and II tumours of the gastro-oesophageal junction).

**Results:**

Tumour necrosis was not significantly associated with any tumour measure other than the degree of differentiation. On multivariate analysis, only age (HR 1.93, 95% CI 1.23–3.04, *P*=0.004), mGPS (HR 2.91, 95% CI 1.51–5.62, *P*=0.001), positive to total lymph node ratio (HR 2.38, 95% CI 1.60–3.52, *P*<0.001) and macrophage infiltration (HR 1.49, 95% CI 1.02–2.18, *P*=0.041) were independently associated with cancer-specific survival in oesophageal adenocarcinoma. Intra-tumoural macrophages were associated with tumour proliferation (*P*<0.001) and CD8+ lymphocytes infiltration (*P*<0.01).

**Conclusion:**

The results of this study suggest that tumour necrosis does not link local and systemic inflammatory responses and is not significantly associated with survival. In contrast, tumour macrophage infiltration appears to have a central role in the proliferative activity and the coordination of the inflammatory cell infiltrate and is independently associated with poorer survival in patients with oesophageal adenocarcinoma.

Oesophageal cancer is the sixth most common causes of cancer death in United Kingdom (Cancer Research UK Information Resource Centre, 2006). Overall survival from oesophageal cancer remains poor even after potentially curative surgery, with or without neo-adjuvant therapy (Lerut *et al*, 2004; Courrech Staal *et al*, 2010).

It is increasingly recognised that outcomes for patients with cancer are determined by host as well as tumour-related factors. Host-related factors include local and systemic inflammatory responses (Colotta *et al*, 2009; McMillan, 2009; Roxburgh and McMillan, 2010; Hanahan and Weinberg, 2011) and an increased systemic inflammatory response before surgery is an independent prognostic factor of survival following resection of oesophageal cancer (Nozoe *et al*, 2001; Ikeda *et al*, 2003; Shimada *et al*, 2003; Gockel *et al*, 2006). For example, on a comprehensive examination of the prognostic value of both tumour- and patient-related factors, only modified Glasgow Prognostic score (mGPS) and positive to total lymph node ratio (LNR) were independent predictor of cancer-specific survival in oesophageal cancer (Dutta *et al*, 2011).

With reference to the local inflammatory response, a pronounced tumour inflammatory cell infiltrate of gastro-oesophageal carcinomas (assessed on haematoxylin and eosin-stained sections) has been reported to be associated with improved survival (Ma *et al*, 1994). It is of interest therefore, that Klintrup *et al* (2005) reported a simplified subjective assessment of the inflammatory infiltrate at the invasive margin of colorectal cancer, including all inflammatory cell types and classifying the infiltrate as low or high grade, had independent prognostic value. This method, in addition to being validated in an independent cohort of colorectal cancer patients (Roxburgh *et al*, 2009a, 2009b), has also been validated in patients with gastro-oesophageal cancer (Crumley *et al*, 2011).

More specifically, CD8+ T-lymphocytes have been reported to provide prognostic information in oesophageal cancer (Schumacher *et al*, 2001; Cho *et al*, 2003). Also, tumour-associated macrophages (CD68+) may have prognostic value in oesophageal cancer (Koide *et al*, 2002; Guo *et al*, 2007). Therefore, it would appear that the type, density and location of tumour inflammatory cells are important in determining cancer outcome in these patients. However, to date the few reports, for example, Crumley *et al* (2011) have been in heterogeneous cohorts of patients including oesophageal, junctional, and gastric sites and squamous and adenocarcinomas. Therefore, the prognostic value of measures of the local inflammatory response in patients with oesophageal cancer, in particular in adenocarcinoma, remains to be established.

The basis of the relationship between the tumour and local and systemic inflammatory responses and outcome is not clear. However, a plausible hypothesis is that rapidly proliferating tumours outgrow their blood supply becoming hypoxic and necrotic thereby stimulating both local and systemic inflammatory responses and angiogenesis that, in turn, promote tumour progression and metastases (Vakkila and Lotze, 2004; Degenhardt *et al*, 2006). Indeed, it has been reported that high tumour proliferative activity is associated with poorer survival in oesophageal cancer (Imdahl *et al*, 2000). Also, Elpek *et al* (2001) have reported that CD34+-positive intra-tumoural microvessel density was associated with poorer survival in oesophageal squamous cell cancer. Finally, consistent with the above hypothesis is the report that tumour proliferative activity was associated with tumour angiogenesis in oesophageal squamous cell cancer (Nomiya *et al*, 2004; Li *et al*, 2008).

Although histological evidence of tumour necrosis is recognised to be associated with decreased survival in other gastrointestinal malignancies such as pancreas and colorectal (Hiraoka *et al*, 2010; Pollheimer *et al*, 2010; Richards *et al*, 2011). To our knowledge, the prognostic of value of tumour necrosis in patients with oesophageal cancer, either squamous or adenocarcinoma, has not been previously reported.

The aim of this study was to examine the relationship between tumour necrosis, tumour proliferation, local and systemic inflammatory responses and microvessel density and survival in patients undergoing potentially curative resection of oesophageal adenocarcinoma.

## Patients and methods

A total of 121 patients undergoing potentially curative resection for oesophageal carcinoma at Glasgow Royal Infirmary between January 1996 and May 2009 were included.

Carcinomas were staged according to the tumour node metastasis (TNM) criteria, sixth edition of the International Union Against Cancer (UICC) Classification (Sobin and Wittekind, 2002). Tumours of the gastro-oesophageal junction were further subdivided by site, using the Siewert classification; type I and II lesions of the gastro-oesophageal junction were designated oesophageal cancers and included in this study, while type III tumours of the cardia were designated gastric cancer and therefore excluded (Siewert and Stein, 1996). Only TNM stage I–III tumours were considered amenable to curative surgical resection and included in the study.

All patients underwent potentially curative en-bloc lymph-adenectomy and survived at least 30 days following surgery. In all, 47 patients received neo-adjuvant chemotherapy, mostly in last 3 years of the study period. Oesophageal cancer patients and type I gastro-oesophageal junctional tumour received two cycles of preoperative cisplatin and 5-flurouracil as in the MRC OE-O2 study (Medical Research Council Oesophageal Cancer Working Group, 2002). Type II gastro-oesophageal junctional tumour was treated with three cycles of ECF (epirubicin, cisplatin and 5-flurouracil).

This study was approved by the Research Ethics Committee of Glasgow Royal Infirmary.

### Biochemical measurements

The coefficient of variation for laboratory measurements of albumin and C-reactive protein, over the range of measurement, was <10% as established by laboratory quality control procedures. The limit of detection of the assay was a C-reactive protein concentration <6 mg l^–1^. A C-reactive protein concentration >10 mg l^–1^ was considered to indicate the presence of a systemic inflammatory response (McMillan *et al*, 2001).

The mGPS was constructed as previously described (McMillan, 2008). An elevated C-reactive protein (>10 mg l^–1^) were assigned a mGPS of 1 or 2 depending on the absence or presence of hypo-albuminaemia (<35 g l^–1^). Patients in whom neither abnormality was present were allocated an mGPS score 0.

### Assessment of tumour necrosis

**Figure 1:**
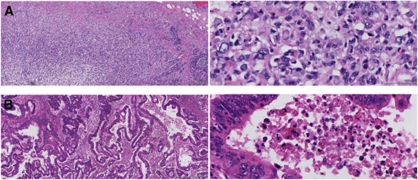
(**A**) Example of absence of necrosis (low-power and high-power view). (**B**) Example of extensive necrosis (low-power and high-power view).

The same routine haematoxylin and eosin slides from the resected tumour specimens were used to evaluate tumour necrosis over the entire area of invasive carcinoma available. The scoring method for evaluating necrosis was adapted from a previously published protocol (Ikpatt *et al*, 2002) where it was subjectively graded into three categories using complete haematoxylin and eosin-stained histological sections. Score 0, absent: no confluent necrosis at all, that is, only single-cell death (apoptosis) identifiable. Score 1, mild: confluent areas of invasive carcinoma cell necrosis in <25% of × 40 fields. Score 2, moderate: confluent areas of invasive carcinoma cell necrosis in 25–50% of × 40 fields. Score 3, extensive: confluent areas of invasive carcinoma cell necrosis in >50% of × 40 fields. These scores were aggregated as low grade (scores 0 and 1) or high grade (scores 2 and 3) (Figure 1A and B, respectively). Confluent necrosis was defined as areas of definite death of small or large foci of carcinoma cells with some or all of the following features: condensation, darker staining, fragmentation or total loss of tumour cell nuclei; increased cytoplasmic eosinophilia, loss of cytological detail, granular eosinophilic debris, occasionally with calcification. All cases were scored independently by two observers (SD, JG) blinded to clinical outcomes. In case of disagreement in the score, an agreed score was determined by revision of the specimen by both of the observers together in a double-headed microscope.

### Assessment of tumour inflammatory infiltrate

**Figure 2:**
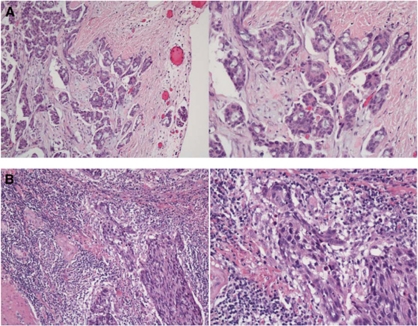
(**A**) Example of ‘low-grade’ local tumour inflammatory infiltrate (low-power and high-power view). (**B**) Example of ‘high-grade’ local tumour inflammatory infiltrate (low-power and high-power view).

The routine haematoxylin and eosin slides from the resected tumour specimens were retrieved from the pathology archive and scored as described by Klintrup *et al* (2005). Tumours were scored based on the appearance at the deepest area of tumour invasion on a four-point score. A score of 0 indicated that there was no increase in the inflammatory cells at the deepest point of the tumours invasive margin; score 1 denoted a mild and patchy increase in the inflammatory cells; score 2 denoted a prominent inflammatory reaction forming a continuous band at the invasive margin with some evidence of destruction of cancer cell islands and score 3 denoted a florid ‘cup-like’ inflammatory infiltrate at the invasive edge with frequent destruction of cancer cell islands. These scores were then subsequently classified as low grade (scores 0 and 1) or high grade (scores 2 and 3) (Figure 2A and B, respectively). All cases were scored independently by two observers (SD or AC and JG). Observers were blinded to the clinical outcome of the patient. The inter-observer intraclass correlation coefficient for tumour inflammatory infiltrate was 0.81.

### Tissue micro array (TMA) construction

**Figure 3:**
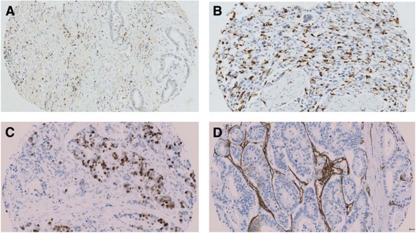
Immunohistochemistry of tissue microarray for CD8+ (**A**); CD68+ (**B**); Ki67 (**C**) and CD34+ (**D**). Positive cells are stained brown. All pictures are in × 200 magnification. The colour reproduction of this figure is available at the *British Journal of Cancer* journal online.

The routine haematoxylin and eosin slides of the resected tumour specimens along with corresponding paraffin blocks were retrieved from the pathology archive for all patients in our study group. A minimum of three representative areas of tumour were defined by the researcher (SD) and the pathologist (JG). Tissue micro arrays were then constructed in triplicate cores 0.6-mm in diameter from each tumour, which were placed in separate TMA blocks (Beecher Scientific, Silver Spring, MD, USA) as previously described (Kononen *et al*, 1998). Sections 2.5 μm thick from each TMA block were mounted on silanised glass slides. These sections were used to perform immunohistochemistry for CD8+ T cells, CD68+ (tumour-associated macrophages), Ki-67 (tumour proliferative index) and CD34+ (for microvessel density) (Figures 3A to D, respectively). This was performed in the Unit of Experimental Therapeutics, Institute of Cancer, College of MVLS University of Glasgow, Western Infirmary, Glasgow, UK.

### Immunohistochemistry

Immunohistochemistry of TMA slides was performed using the Dako Envision method (Dako, Cambridgeshire, UK). The primary antibody for CD8+ was monoclonal mouse anti-human CD8+, clone CD8+/144B (DAKO, Glostrup, Denmark) at a dilution of 1 : 100 (overnight incubation) and for CD68+ was monoclonal mouse anti-human CD68+, clone PG-M1 (DAKO) at a dilution of 1 : 200 (1-h incubation). The primary antibody for Ki-67 was monoclonal mouse anti-human Ki-67, clone MIB-1(DAKO) at a dilution of 1 : 50 (overnight incubation) and for CD34+ was monoclonal mouse anti-human, CD34+ class II, clone QBEnd 10 (DAKO) at a dilution of 1 : 150 (30-min incubation).

Cores were dewaxed and rehydrated. Antigen retrieval was performed by keeping the slides in Tris EDTA buffer (pH 8), in pressure cooker for 5 min. Endogenous peroxidise was blocked by incubation in 3% hydrogen peroxide for 10 min. The cores were then incubated with the normal horse serum at dilution 1 : 20 for 20 min at 25 °C to block nonspecific binding sites. Respectively primary antibody was added in appropriate concentrations. Sites of binding were detected using the Envision technique (DAKO code K5007) with DAB (3-3′-diaminobenzidine, Vector code SK 4001, Burlingame, CA, USA), a chromogenic substrate, according to the manufacturer's instruction. Cores were counterstained with haematoxylin, dehydrated and mounted with DPX. Appropriate positive controls were included in each run and negative controls were omission of the primary antibody.

### Morphometry

For automated image analysis of digitised slides were accessed through the Slidepath Image Analysis system (Leica Microsystems GmbH, Wetzlar, Germany) and evaluated with the program's nuclear (for Ki-67), cytoplasmic (for CD68+) and membranous (for CD8+) scoring algorithm.

Individual TMA cores were identified, annotated on the scanned image and associated with TMA map entries. Individual nuclei stained with haematoxylin and/or polymerised diaminobenzidine are identified by a thresholding and segmentation algorithm, which outlines nuclei and separates touching nuclei. Nuclear size (area) limits can be specified to accept or reject individual nuclei to be quantified. Staining for Ki-67 in each nucleus was classified as positive or negative based on the threshold specified by the observer. Pseudo-colours (red/orange/yellow/blue) display these staining intensity measurements for individual nuclei, allowing thresholds to be chosen appropriately. These thresholds were chosen using a sample of TMA cores from the whole cohort and once chosen were used for analysis over the entire patient cohort without further adjustment.

For CD68+, intracellular and positive pixel detection algorithm was used and for the CD8+, algorithm for thin cell membrane was used. Each TMA cores were crosschecked by one of the investigators (SD) to detect any obvious error. Moreover, at least 25% of the cases were manually scored to produce inter-observer reproducibility. The inter-observer intraclass correlation coefficient values for Ki67, CD68+ and CD8+ were 0.93, 0.87 and 0.74, respectively.

To assess intra-tumoural microvessel density, immunohistochemical staining for CD34+ was performed. Quantitative assessment of CD34+ was performed by manual counting of CD34+-positive endothelial cells or cluster regardless of whether a vessel lumen was seen in each of the TMA cores. All cores were counted by one of us (SD) and at least 25% of the cases were counted by a second observer (ZM) and interclass correlation coefficient for CD34+ was 0.83.

### Statistical analysis

Survival analysis of the group variables was performed using the Cox proportional hazard model including deaths up to the end of May 2011. Multivariate survival analysis, including all covariates with a *P*-value of ⩽0.1 was performed using a stepwise backward procedure to derive a final model of the variables that had a significant independent relationship with survival. To remove a variable from the model, the corresponding *P*-value had to be >0.05. The relationships between the mGPS and other variables were analysed using the Mantel–Haenszel (*χ*^2^) test for trend as appropriate. Analysis was performed using SPSS software (SPSS Inc., Chicago, IL, USA).

## Results

A total of 121 patients with oesophageal cancer were included in our study (Table 1). Overall, the majority of patients were male (81%), <65 years of age (60%) and had an mGPS of 0 (87%). Most of the patients had pTNM stage II or III (82%), adenocarcinoma (81%), well to moderately differentiated tumour (59%), no resection margin involvement (79%), lymph node involvement (72%) and had high-grade tumour necrosis (53%). In all, 27 patients had high-grade peri-tumour inflammatory infiltrate according to Klintrup–Makinen criteria (22%). The median values for CD8+, CD68+ and Ki-67 were 4.7%, 14.5% and 20.3%, respectively, and for CD34+ the median value was 40. The majority did not receive either neo-adjuvant (61%) or adjuvant (85%) therapy.

The relationship between tumour type (adeno- and squamous carcinomas) and clinico-pathological characteristics are shown in Table 1. Patients with oesophageal adenocarcinoma were more likely to be male (*P*<0.001), had higher infiltration of CD8+ (*P*<0.001) and CD68+ (*P*<0.01), had higher TNM stage (*P*<0.05), had neo-adjuvant therapy (*P*<0.01), and as well as had higher CD34+-positive microvessel compared with squamous cell carcinoma (*P*<0.001, Table 1). There was no significant difference in the degree of tumour necrosis.

Owing to the limited number of patients with squamous cancer the relationship between clinic-pathological factors and cancer-specific survival was examined only in those patients with oesophageal adenocarcinomas (*n*=98, Tables 2). In patients with oesophageal adenocarcinoma, the median follow-up of survivors was 45 months with a minimum of 22 months. During this period, 49 (50%) patients died of their cancer and 4 (4%) patients died of non-cancer causes. In patients with oesophageal squamous cell carcinoma, median follow-up of survivors was 90 months with a minimum of 23 months. During this period, 11 (48%) patients died of their cancer and 4 (17%) patients died of non-cancer causes.

**Figure 4:**
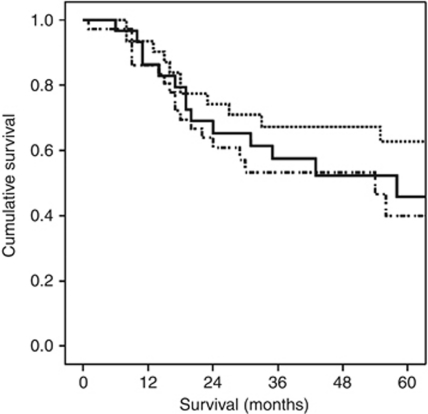
The relationship between tumour CD68+ infiltration (top to bottom, tertiles 1/2/3) and cancer-specific survival in patients undergoing resection for oesophageal adenocarcinoma.

On univariate analysis, only age (*P*<0.05), mGPS (*P*<0.01), TNM stage (*P*⩽0.001), tumour differentiation (*P*⩽0.001), resection margin (*P*<0.10), LNR (*P*<0.001), Klintrup–Makinen score (*P*<0.05), CD8+ (*P*<0.05), CD68+ (*P*<0.10) and Ki-67 (*P*<0.05) were significantly associated with cancer-specific survival. On multivariate analysis, age (HR 1.93, 95% CI 1.23–3.04, *P*=0.004), mGPS (HR 2.91, 95% CI 1.51–5.62, *P*=0.001), LNR (HR 2.38, 95% CI 1.60–3.52, *P*<0.001) and CD68+ (HR 1.49, 95% CI 1.02–2.18, *P*=0.041, Figure 4) retained independent significance (Table 2).

Interrelationships between clinical and pathological characteristics are shown in Table 3. Male sex was associated with poor tumour differentiation (*P*<0.01). Tumour node metastasis stage was directly associated with poor tumour differentiation (*P*<0.01), positive resection margin (R1) (*P*<0.001) and LNR (*P*<0.001). Poorly differentiated oesophageal adenocarcinoma was directly associated with a LNR (*P*<0.001), inversely with the necrosis score (*P*<0.05) and directly with CD68+ infiltration (*P*<0.05). Positive resection margin (R1) was directly associated with a LNR (*P*<0.01) and inversely with CD8+ infiltration (*P*<0.01). A LNR was directly associated with the Klintrup–Makinen score (*P*<0.01). The Klintrup–Makinen score was directly associated with CD8+ infiltration (*P*<0.01). Tumour necrosis was not significantly associated with any tumour measure other than the degree of differentiation. Tumour CD8+ infiltrate was directly associated with CD68+ infiltration (*P*<0.01), CD34+ (*P*<0.05) and neo-adjuvant therapy (*P*<0.05). Tumour CD68+ infiltration of the oesophageal adenocarcinoma was directly associated with the Ki-67 proliferation index (*P*<0.001) as well as neo-adjuvant therapy (*P*<0.01). The tumour Ki-67 proliferation index was directly associated with CD34+ microvessel density (*P*=0.05) and neo-adjuvant therapy (*P*<0.001).

Interrelationships between clinical and pathological characteristics of patents who did not receive neo-adjuvant chemotherapy in oesophageal adenocarcinoma are shown in Table 4 (*n*=53). Tumour node metastasis stage was directly associated with poor tumour differentiation (*P*<0.05), positive resection margin (R1) (*P*=0.001) and LNR (*P*<0.001). Poorly differentiated oesophageal adenocarcinoma was directly associated with a LNR (*P*=0.001) and directly with CD68+ infiltration (*P*<0.05). Positive resection margin (R1) was directly associated with a LNR (*P*<0.05) and inversely with CD8+ infiltration (*P*<0.05). A LNR was directly associated with the Klintrup–Makinen score (*P*<0.01) and inversely with CD8+ infiltration (*P*<0.05). The Klintrup–Makinen score was directly associated with CD8+ infiltration (*P*<0.05).

The relationship between clinic-pathological factors and cancer-specific survival was examined also in a subgroup of oesophageal adenocarcinoma patients who did not receive neo-adjuvant therapy (*n*=53, Tables 5). On univariate analysis, only age (*P*<0.05), mGPS (*P*⩽0.001), TNM stage (*P*<0.01), tumour differentiation (*P*<0.05), resection margin (*P*<0.05), LNR (*P*<0.001), Klintrup–Makinen score (*P*<0.05) and CD68+ (*P*<0.10) were significantly associated with cancer-specific survival. On multivariate analysis, age (HR 2.62, 95% CI 1.27–5.39, *P*=0.009), mGPS (HR 12.71, 95% CI 4.15–38.94, *P*<0.001), LNR (HR 3.18, 95% CI 1.77–5.72, *P*<0.001) and CD68+ (HR 1.88, 95% CI 1.12–3.15, *P*=0.017) retained independent significance (Tables 5).

When multivariate survival analysis was carried out in those patients with stage III adenocarcinoma alone (*n*=50), only age (HR 1.90, 95% CI 1.14–3.17, *P*=0.014) and mGPS (HR 2.91, 95%CI 1.12–7.58, *P*=0.029) were independently associated with cancer-specific survival. None of the markers of inflammatory cell infiltrate, including CD68+ were associated with cancer-specific survival.

## Discussion

The results of this study show that, in patients with oesophageal cancer, the extent of the inflammatory infiltrate and angiogenesis was greater in adenocarcinoma compared with squamous carcinoma. Furthermore, within the adenocarcinoma cohort, although tumour necrosis was not significantly associated with proliferative activity, inflammatory cell infiltrate and angiogenesis, tumour proliferative activity was directly associated with the extent of macrophage infiltration. Also, the generalised inflammatory and cytotoxic T-lymphocyte infiltrate were inversely associated with the LNR and resection margin respectively. Moreover, patients with neo-adjuvant therapy had more intra-tumoural inflammatory cell infiltrate as well as had higher proliferative activity. When the prognostic value of such tissue factors were examined, only the tumour macrophage infiltration retained significance. Taken together, these results would suggest that tumour necrosis does not link local and systemic inflammatory responses but that the tumour inflammatory infiltrate, in particular macrophages, have a role in the control of tumour progression and dissemination in patients with oesophageal adenocarcinoma.

The present report of tumour necrosis in oesophageal adenocarcinoma is to our knowledge unique in the literature and therefore further work is required to establish whether it has prognostic value and relationship with patient- and tumour-related factors in oesophageal cancer. Furthermore, the basis of the differences in the inflammatory cells and microvessel density in squamous and adenocarcinomas is not clear. However, it may reflect more aggressive nature of squamous cell carcinoma (Siewert *et al*, 2001; Stein *et al*, 2005). Irrespective, the results do emphasise the heterogeneous nature of oesophageal cancer and may have been a significant confounding factor in previous studies.

Given that preoperative neo-adjuvant chemotherapy might influence the some of the clinico-pathological characteristics examined in this study, a subgroup analysis was performed on patients who did not receive neo-adjuvant therapy. Indeed, when compared with the no neo-adjuvant therapy group, the neo-adjuvant therapy group had higher Ki-67 proliferation index (*P*<0.001) and higher densities of both CD8+ T-lymphocytes (*P*=0.016) and CD68+ macrophages (*P*=0.004). In contrast, in the no neo-adjuvant therapy group alone, the association between higher Ki-67 proliferation index and densities of both CD8+ T-lymphocytes and CD68+ macrophages was not significant. The basis of this apparent contradiction is not clear, however, it may suggest that neo-adjuvant therapy does influence the relationship between Ki-67 proliferation index and densities of both CD8+ T-lymphocytes and CD68+ macrophages.

The role of Ki67 in oesophageal cancer is also not well established. It has been reported to predict the complete response in oesophageal cancer following chemo-radio therapy (Takeuchi *et al*, 2003; Ressiot *et al*, 2008). However, there are some conflicting reports on the predictive value of Ki67 on survival in oesophageal squamous cell cancer (Youssef *et al*, 1995; Sarbia *et al*, 1996; Imdahl *et al*, 2000). In this study, a high proliferation index in oesophageal adenocarcinoma was associated with poor outcome (on univariate survival analysis) and was also associated with intra-tumoural macrophage infiltration (CD68+) and microvessel density (CD34+).

With reference to the tissue factors examined, the results of this study are consistent with previous literature. Cho *et al* (2003) have reported the prognostic value of CD8+ lymphocyte in 122 patients with oesophageal squamous cell carcinoma. Similarly, Tsuchikawa *et al* (2011) reported the prognostic significance of CD8+ lymphocytes in 98 patients with oesophageal squamous cell carcinoma. In contrast, in another study involving 130 patients with oesophageal adenocarcinoma, CD8+ lymphocyte was not associated with survival (Zingg *et al*, 2010). Also, that tumour-associated macrophages (CD68+) had prognostic value in oesophageal cancer (Koide *et al*, 2002; Guo *et al*, 2007). Indeed, Koide *et al* (2002) reported, in 56 patients, that the CD68+ macrophage infiltrate was associated with tumour proliferation index (Ki67) and disease progression in oesophageal squamous cell cancer. Also, Guo *et al* (2007) reported that, 137 patients with oesophageal squamous carcinoma, a high tumour macrophage infiltrate and a low lymphocytic infiltrate were associated with poor outcome. However, that the high tumour macrophage infiltrate had superior prognostic value. In this study, the relationships between tumour necrosis, tumour proliferation, intra-tumoural inflammatory cell infiltrates and microvessel density and survival in patients undergoing potentially curative resection of oesophageal adenocarcinoma was examined. Given that, of the tumour inflammatory cell infiltrate, the macrophages were most closely associated with survival it would be of interest to examine the phenotype (e.g., M1 or M2) of the macrophages in detail in patients with oesophageal adenocarcinoma.

In the context of the present results, it is also important to acknowledge that, there is some recent evidence that CD68+ can be expressed on non-myeloid cells including carcinomas (Gottfried *et al*, 2008). Indeed, Gottfried *et al* (2008) concluded that CD68+ is not a selective macrophage marker but rather a lysosomal protein that is enriched in macrophages. In this study, the morphology of the cell type was clearly considered during the assessment of CD68+ expression and, although unlikely, it is conceivable that the results of this study may be have been influenced by expression of CD68 by non-macrophages cells. Further work is required to examine the extent of possible confounding in this study.

It was of interest therefore, in this study, that CD68+ macrophages was strongly associated with CD8 + T lymphocytes and tumour proliferative index (Ki67) in oesophageal adenocarcinoma. Taken together with the other results above, this would suggest that high tumour lymphocytic infiltrate prevents tumour progression and a high tumour macrophage infiltration promotes tumour proliferation. Given that they are both generally increased together it is likely that the balance of these inflammatory cells determines whether there is tumour progression. However, more work is required to be undertaken to better define these relationships in oesophageal adenocarcinoma.

In summary, of the tissue-based factors examined in this study tumour macrophage infiltration appeared to have a central role in the proliferative activity and the coordination of the inflammatory cell infiltrate and was independently associated with poor outcome in patients with oesophageal adenocarcinoma.

## Figures and Tables

**Table 1:** Clinico-pathological characteristic of patients undergoing potentially curative resection for oesophageal cancer (*n*=121)

	**Adeno (*n*=98)**	**Squamous (*n*=23)**	***P*-value**
*Patient-related factors*			
Age (<65/65–74/⩾75 years)	57/35/6	15/6/2	0.755
Sex (male/female)	83/15	10/13	<0.001
mGPS (0/1/2)	87/9/2	18/5/0	0.362
Klintrup–Makinen score (low grade/high grade)	77/21	17/6	0.591
CD8+ tertiles (1/2/3)	25/39/34	17/5/1	<0.001
CD68+ tertiles (1/2/3)	25/35/38	12/9/2	0.002
			
*Tumour-related factors*			
TNM stage (I/II/III)	15/33/50	7/11/5	0.011
Tumour differentiation (well-mod/poor)	60/38	11/12	0.251
Resection margin (R0/R1)	75/23	20/3	0.399
LNR (0/⩽ 0.2/>0.2)	35/35/28	11/9/3	0.132
Necrosis score (low grade/high grade)	48/50	9/14	0.488
Ki67 tertiles (1/2/3)	34/29/35	5/8/10	0.284
CD34+ tertiles (1/2/3)	26/33/39	15/8/0	<0.001
Neo-adjuvant therapy (no/yes)	53/45	21/2	0.001
Adjuvant therapy (no/yes)	81/17	22/1	0.191
			
Alive	45	8	
Cancer-specific death	49	11	
Non-cancer death	4	4	0.893^a^

Abbreviations: LNR=lymph node ratio; mGPS=modified Glasgow Prognostic score; TNM=tumour node metastasis.

a Log rank test (Mantel–Cox).

**Table 2:** Relationships between clinic-pathological factors and cancer-specific survival, in patients selected for potentially curative resection for oesophageal adenocarcinoma (*n*=98)

	**Univariate analysis**		**Multivariate analysis**	
	**Hazard ratio (95% CI)**	***P*-value**	**Hazard ratio (95% CI)**	***P*-value**
*Patient-related factors*				
Age (<65/65–74/⩾75 years)	1.69 (1.11–2.59)	0.016	1.93 (1.23–3.04)	0.004
Sex (male/female)	0.47 (0.17–1.31)	0.148		
mGPS (0/1/2)	2.39 (1.36–4.18)	0.002	2.91 (1.51–5.62)	0.001
				
*Tumour-related factors*				
TNM stage (I/II/III)	2.27 (1.42–3.64)	0.001		0.345
Tumour differentiation (well-mod/poor)	2.52 (1.43–4.44)	0.001		0.202
Resection margin (R0/R1)	1.80 (0.99–3.29)	0.053		0.805
Positive to total lymph node ratio (0/⩽0.2/>0.2)	2.47 (1.71–3.58)	<0.001	2.38 (1.60–3.52)	<0.001
Neo-adjuvant therapy (no/yes)	1.39 (0.74–2.61)	0.304		
Adjuvant therapy (no/yes)	1.74 (0.83–3.65)	0.141		
				
Klintrup–Makinen score (low/high grade)	0.35 (0.15–0.82)	0.016		0.076
Necrosis score (low/high grade)	1.12 (0.64–1.97)	0.695		
CD8 tertiles	0.69 (0.48–0.99)	0.048		0.697
CD68 tertiles	1.38 (0.99–1.94)	0.061	1.49 (1.02–2.18)	0.041
Ki67 tertiles	1.46 (1.01–2.12)	0.048		0.479
CD34 tertiles	0.94 (0.67–1.34)	0.736		

Abbreviations: CI=confidence interval; LNR=lymph node ratio; mGPS=modified Glasgow Prognostic score; TNM=tumour node metastasis.

**Table 3:** Interrelationships between different pathological and clinical parameters in patients selected for potentially curative resection for oesophageal adenocarcinoma (*n*=98)

	**Sex**	**mGPS**	**TNM stage**	**Tumour differentiation**	**Resection margin**	**LNR**	**Klintrup– Makinen score**	**Necrosis score**	**CD8 tertiles**	**CD68 tertiles**	**Ki67 tertiles**	**CD34 tertiles**	**Neo-adjuvant therapy**
Age in years (<65/65–74/⩾75 years)	0.199	0.248	0.688	0.939	0.991	0.586	0.977	0.505	0.510	0.749	0.110	0.923	0.639
Sex (male/female)		0.155	0.605	0.008	1.00	0.170	0.302	0.091	0.609	0.203	0.813	0.693	0.400
mGPS			0.823	0.616	0.569	0.328	0.894	0.228	0.243	0.838	0.311	0.723	0.988
TNM stage (I/II/III)				0.008	<0.001	<0.001	0.403	0.754	0.203	0.593	0.430	0.081	0.420
Tumour differentiation (well-mod/poor)					1.00	<0.001	0.322	0.012	0.422	0.024	0.655	0.922	0.838
Resection margin (R0/R1)						0.004	0.144	0.344	0.004	0.362	0.307	0.719	0.242
LNR							0.009	0.388	0.093	0.332	0.293	0.168	0.417
Klintrup–Makinen score (low/igh grade)								1.00	0.002	0.078	0.105	0.813	1.00
Necrosis score (low/high grade)									0.219	0.724	0.140	0.109	0.839
CD8 tertiles										0.002	0.051	0.044	0.016
CD68 tertiles											<0.001	0.994	0.004
Ki67 tertiles												0.050	<0.001
CD34													0.261

Abbreviations: LNR=lymph node ratio; mGPS=modified Glasgow Prognostic score; TNM=tumour node metastasis.

**Table 4:** Interrelationships between different pathological and clinical parameters in patients selected for potentially curative resection for oesophageal adenocarcinoma without neo-adjuvant therapy (*n*=53)

	**Sex**	**mGPS**	**TNM stage**	**Tumour differentiation**	**Resection margin**	**LNR**	**Klintrup– Makinen score**	**Necrosis score**	**CD8 tertiles**	**CD68 tertiles**	**Ki67 tertiles**	**CD34 tertiles**
Age in years (<65/65–74/⩾75 years)	0.131	0.262	0.706	0.978	0.674	0.412	0.991	0.266	0.951	0.753	0.375	0.952
Sex (male/female)		0.545	0.654	0.070	0.706	0.259	0.416	0.302	0.615	0.580	0.433	0.726
mGPS			0.624	0.645	0.988	0.085	0.697	0.833	0.661	0.440	0.454	0.806
TNM stage (I/II/III)				0.034	0.001	<0.001	0.325	0.380	0.169	0.229	0.503	0.183
Tumour differentiation (well-mod/poor)					1.00	0.001	0.503	0.053	0.906	0.028	0.740	0.849
Resection margin (R0/R1)						0.011	0.149	0.074	0.032	0.347	0.789	0.726
LNR							0.008	0.921	0.042	0.076	0.198	0.318
Klintrup–Makinen score (low/high grade)								0.509	0.011	0.722	0.160	0.412
Necrosis score (low/high grade)									0.485	0.359	0.051	0.685
CD8 tertiles										0.083	0.570	0.799
CD68 tertiles											0.259	0.709
Ki67 tertiles												0.507

Abbreviations: LNR=lymph node ratio; mGPS=modified Glasgow Prognostic score; TNM=tumour node metastasis.

**Table 5:** Relationships between clinico-pathological factors and survival, in patients selected for potentially curative resection for oesophageal adenocarcinoma without neo-adjuvant therapy (*n*=53)

	**Univariate analysis**		**Multivariate analysis**	
	**Hazard ratio (95% CI)**	***P*-value**	**Hazard ratio (95% CI)**	***P*-value**
*Patient-related factors*				
Age (<65/65 –74/⩾75 years)	2.07 (1.13–3.77)	0.018	2.62 (1.27–5.39)	0.009
Sex (male/female)	0.77 (0.27–2.21)	0.625		
mGPS (0/1/2)	4.14 (1.80–9.51)	0.001	12.71 (4.15–38.94)	<0.001
				
*Tumour-related factors*				
TNM stage (I/II/III)	2.46 (1.34–4.49)	0.004		0.246
Tumour differentiation (well-mod/poor)	2.35 (1.13–4.92)	0.023		0.552
Resection margin (R0/R1)	2.13 (1.02–4.49)	0.046		0.607
Positive to total lymph node ratio (0/⩽0.2/>0.2)	2.45 (1.55–3.89)	<0.001	3.18 (1.77–5.72)	<0.001
Adjuvant therapy (no/yes)	2.45 (0.83–7.22)	0.105		
				
Klintrup–Makinen score (low/high grade)	0.28 (0.08–0.92)	0.036		0.055
Necrosis score (low/high grade)	1.29 (0.61–2.70)	0.505		
CD8 tertiles	0.68 (0.43–1.10)	0.117		
CD68 tertiles	1.49 (0.97–2.28)	0.066	1.88 (1.12–3.15)	0.017
Ki67 tertiles	1.35 (0.78–2.35)	0.289		
CD34 tertiles	0.73 (0.45–1.20)	0.214		

Abbreviations: CI=confidence interval; LNR=lymph node ratio; mGPS=modified Glasgow Prognostic score; TNM=tumour node metastasis.
